# Recent Developments in Thermally Stable Transparent Thin Films for Heater Applications: A Systematic Review

**DOI:** 10.3390/nano14242011

**Published:** 2024-12-14

**Authors:** Worawat Traiwattanapong, Vandana Molahalli, Apichart Pattanaporkratana, Nattaporn Chattham

**Affiliations:** 1 Department of Physics, Faculty of Science, Kasetsart University, Bangkok 10900, Thailand; worawat.tra@ku.th (W.T.); fsciacp@ku.ac.th (A.P.); 2 Department of Physics, B.M.S. College of Engineering, Bull Temple Road, Bengaluru 560019, India; vandanam254@gmail.com; 3 Centre for Nano-Materials and Displays, B.M.S. College of Engineering, Bull Temple Road, Basavanagudi, Bengaluru 560019, India

**Keywords:** transparent thin films, thermal heaters, metal oxides, flexibility and stability, mechanical and optical properties

## Abstract

Transparent thin-film heaters have sparked great interest in both the scientific and industrial sectors due to their critical role in various technologies, including smart windows, displays, actuators, and sensors. In this review, we summarize the structural design, fabrication methods, properties, and materials used in thin-film heaters. We also discuss methods to improve their efficiency and recent advancements in the field, and provide insights into the market size, growth, and future outlook for thin-film heaters.

## 1. Introduction

Electrically conductive layers are present in transparent heaters (THs), which are transparent to the naked eye. The Joule effect causes the transparent heater to produce heat when an electrical current passes over it [1,2]. Numerous gadgets may effectively exploit this heat. As a result, THs are used in a wide range of applications, and the corresponding market for THs is expanding quickly [3]. This market includes a wide range of devices for many industrial sectors, including transportation, buildings, healthcare, and sports, as well as various displays, sensors, deicers, and defoggers [2]. For example, THs can raise the temperature for anti-fogging systems [4], anti-icing, and deicing of optics [5] and optical displays (particularly helpful for transportation), or they can supply the warmth needed to extend the operating temperature of liquid crystal displays (LCDs) in cold situations [6].

Although thin-film fabrication has been widely documented for use in a variety of real-world applications, including photovoltaics, batteries, LEDs, electronic semiconductor devices, and even pharmaceutical applications, the first studies involving measurements of thin-film thermal conductivity were initiated in 1973 by Prem Nath and K. L. Chopra, and in 1988 by David G. Cahill at Cornel University [7]. Connell and his colleagues did not reveal the function of carbon- and polymer-based thin films and ribbons as highly thermally conductive materials until 2006. Sun’s group reported record-breaking anisotropic thermal conductivities for carbon nanosheet–polymer composites three years later, in 2009 [8]. After that, the use of thermally conductive thin films for electronic thermal management applications gained momentum and grew to become a popular field of study worldwide [9,10]. Even though the synthesis of thermally conductive thin films has been the subject of numerous research studies to date, and their potential for thermal management applications has been assessed, this is still a relatively new and developing field of study that holds great potential for advancement. The study of thermally conductive sheets and films has grown significantly over the last 20 years. As a result, several novel opportunities have emerged that can influence the direction of the electronics sector [11].

The heat response period, steady-state temperature, homogeneity, mechanical properties (under stretching test), cycling/thermal/electrical/environmental stability including aging, electrical, and optical properties [12,13], the TH’s fabrication process (with temperature becoming a potential problem for some applications), the material used, the size of the TH, and, finally, the total cost of fabrication, including materials and processes, are the main scientific and technological features associated with thermoplastic hybrid heaters [2].

**Figure 1:**
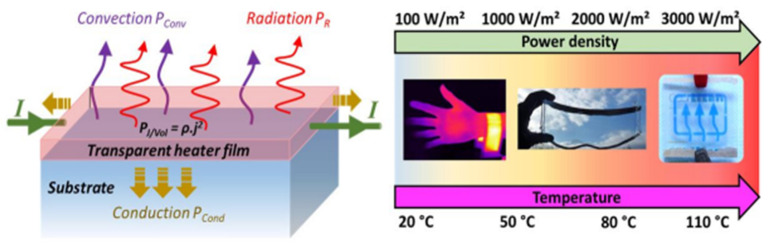
Transparent heater film [11].

Furthermore, the quality of the transparent conductive layer has a direct impact on the thermal reaction time, stable heating temperature, operating voltage, temperature uniformity, and cycling stability of TFHs [14]. Due to their low resistivity and high optical transmittance, Sn-doped In_2_O_3_ (ITO) or F-doped SnO_2_ (FTO) films made by chemical vapor deposition or sputtering have been used up to now as the transparent conductive layer in commercial transparent TFHs [15]. Despite the low resistance and high transmittance of both ITO and FTO films, their use as flexible and transparent electrodes for flexible and transparent TFHs is severely hampered by the brittleness of the oxide-based transparent conductive layer. Furthermore, it is thought that the high production temperature for the FTO film and the high cost of indium for the ITO film represent a constraint for oxide-based transparent conductive electrodes [16]. Many transparent conductive materials, including conducting polymers, metal-based electrodes (Ag nanowires, Ag grids, and Cu nanowires), carbon-based electrodes (carbon nanotube, graphene, and graphene oxides), and hybrid electrodes [17], have been proposed as the transparent conductive layer for high-performance TFHs in place of conventional ITO and FTO films shown in Figure 1 [18].

To achieve a high heating power density and enable large-area applications, the ideal TH should possess the following qualities: (a) low sheet resistance; (b) high resistance uniformity; (c) high transparency in the visible; (d) fast heating speed and high attainable temperature, which are dependent on the thermal characteristics of both the heating film and the substrate; (e) high transparency in the visible arena and excellent mechanical, chemical, and environmental stability; (f) inexpensive material and fabrication costs; and (g) a maximum temperature that self-limits, removing the need for extra control circuits. We will go over the most recent developments and techniques in this review to improve the properties, integration, and stability of these innovative THs.

**Figure 2:**
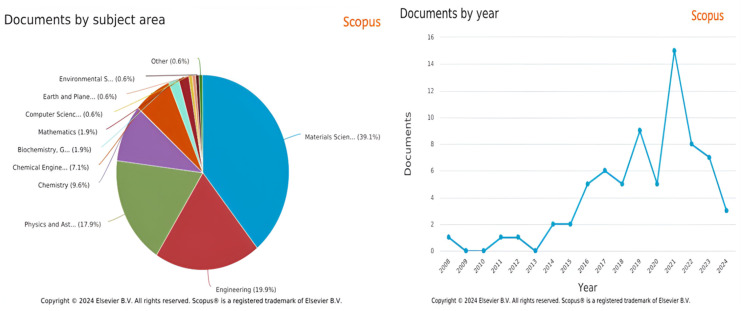
Web of Science data: number of publications in the last 15 years and documents by subject area in percentage.

Based on the Web of Science data shown in Figure 2, only 14-to-16 documents were published in the last seven years. This is much less compared to other fields. Therefore, the researcher has to give more attention to the transparent thin film for heater applications.

## 2. Structural Design of Thin-Film Heaters (TFHs)

Compact and effective, thin-film heaters are frequently found in industrial, medicinal, and electronic applications. A thin-film heater’s structural design carefully takes into account its material, thermal, and functional properties. For thin-film heaters tailored for particular uses, this structural design framework guarantees a balance between efficiency, dependability, and manufacturing feasibility.

**Figure 3:**
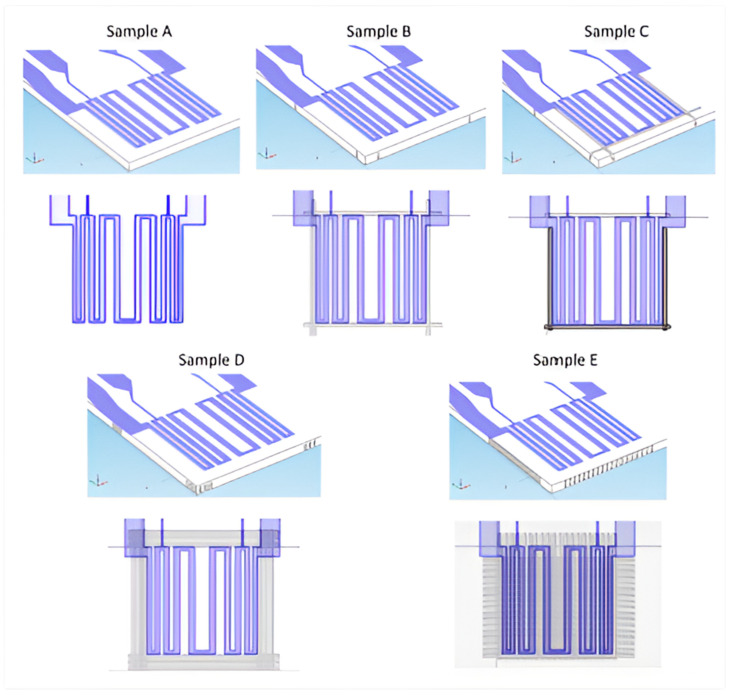
The top image of the glass-based thin-film heater simulation structures. Sample A serves as the reference. Sample B has four linear trenches on the glass back side, Sample C has three linear trenches on the glass front side and one on the back side, and Sample E’s trenches form a chessboard geometry underneath the heater. Sample D has three concentric rectangular trenches cut on the back side. The pad design has been selected to contribute to a low series resistance [19].

The reference is shown in Figure 3, Sample A. It is constructed from a 5 cm × 2.5 cm × 1 mm glass substrate with a thin-film heater shaped like a chirped serpentine on top. This ensures good temperature uniformity throughout the microheater’s surface, but especially in the “active area”, which is the space between the heater’s two voltage taps in its four-terminal geometry. The film has a thickness of Cr/1500 nm, or an Al/300 nm-thick Cr stacked structure. Differently shaped mechanical tunnels have been envisioned in the glass bulk surrounding the heater in order to maximize the thermal behavior of the entire construction.

In Sample B, a rectangular trench is taken into consideration on the back side, which is the opposite side of the heater. It is 240 µm wide and 800 µm deep, with the remaining glass being only 200 µm thick in accordance with the trench. It is placed completely around the heater at an 80 µm distance from the heater edge. Since the total width of the resulting trenches in glass is around 240 µm and the normal thickness of spinning sews in dicing machines is 200 µm, we took this into consideration. The trench in Sample C is on the same glass side (front side) of the heater as Sample B, but it has the same layout to prevent severing the metal electrodes.

The trenches in D and E were stopped at a distance of roughly 80 µm from the opposing surface, so that they are the same width (240 µm) and depth (920 µm). There was 240 µm between parallel trenches. The heater’s outer border and the trench’s edge have to be at least 80 m apart. The other geometrical features result from the technique used to construct the trenches, while the implemented trench depth takes into consideration practical considerations pertaining to the fragility of the glass substrate. The electrical and thermal properties of the glass substrate and the metallic resistor are included in the simulations [19].

## 3. Fabrication Method and Cost Efficiency of TFHs

Thin-film heaters (TFHs) are made by fusing materials with high electrical conductivity and transparency like graphene, metal nanowires, or conductive polymers with flexible plastic or glass substrates. Maximizing optical transmittance, guaranteeing consistent heating, and balancing production costs are important factors.

ITO can be sputtered or chemically vapor-deposited onto a transparent surface, such as glass or PET. It provides low sheet resistance and high optical transparency. However, it is costly, brittle, and unsuitable for flexible electronics because the limited availability of materials and intricate deposition methods make ITO production comparatively expensive [20].

Materials such as copper nanowires (CuNWs) or silver nanowires (AgNWs) are used in metal nanowire coatings. These coatings are applied by roll-to-roll printing of nanowire dispersions, spin coating, or spray coating. Flexibility, openness, and cost-effectiveness at scale are some of their benefits. For durability, protective coatings are necessary since copper nanowires are very vulnerable to oxidation. Nanowire coatings are less expensive than indium tin oxide (ITO), particularly for flexible applications [21].

Chemical vapor deposition (CVD) is used to generate graphene sheets on substrates such as copper and then transfer them to transparent substrates to create graphene-based heaters. These heaters are very flexible and have good thermal and electrical conductivity [22].

But at the moment, the CVD and transfer procedures are costly and demand for specialized tools. Although process modification may be able to lower costs, graphene-based heaters are currently more expensive than metal nanowires. Conductive polymer coatings are applied by spin coating or inkjet printing and contain components such as PEDOT:PSS [23]. These coatings are printable, flexible, and transparent. However, in certain applications their effectiveness is limited due to their poorer conductivity compared to graphene or ITO. In spite of this disadvantage, conductive polymer coatings are scalable and reasonably priced, which makes them a viable choice for some applications. Techniques including roll-to-roll processing, inkjet printing, and the use of inexpensive materials like copper or carbon nanotubes are advised for economical fabrication. For applications that need lightweight and bending designs, flexible substrates like PET or polycarbonate are also favored over glass.

The optimum performance-to-cost ratio is provided by metal nanowires, conductive polymers, and scalable production processes like roll-to-roll processing, particularly for flexible and large-scale TFH applications [24].

The most promising methods for producing TFHs at a reasonable cost are roll-to-roll manufacturing, conductive polymers, and metal nanowires. Particularly for applications needing flexibility and lightweight designs, these methods provide a compromise between performance, scalability, and price.

## 4. Principal and Experimental Study of THs

The primary characteristics of THs and the experimental techniques used to study them are intended to be introduced in this section. Generally speaking, a TH is deemed efficient if it exhibits low electrical resistance, good stability, a low haze factor, high optical transmittance, at least in the visible region, and a regulated heating rate. First, we will concentrate on optical transparency and electrical resistance. Next, we will go over how to look into a thermal heater (TH)’s main operating modes and heating performances, which are specifically examined using temperature readings at various length scales. This allows us to measure distinguishing characteristics such as the heating rate and the temperature–areal heat power density relationship.

**Figure 4:**
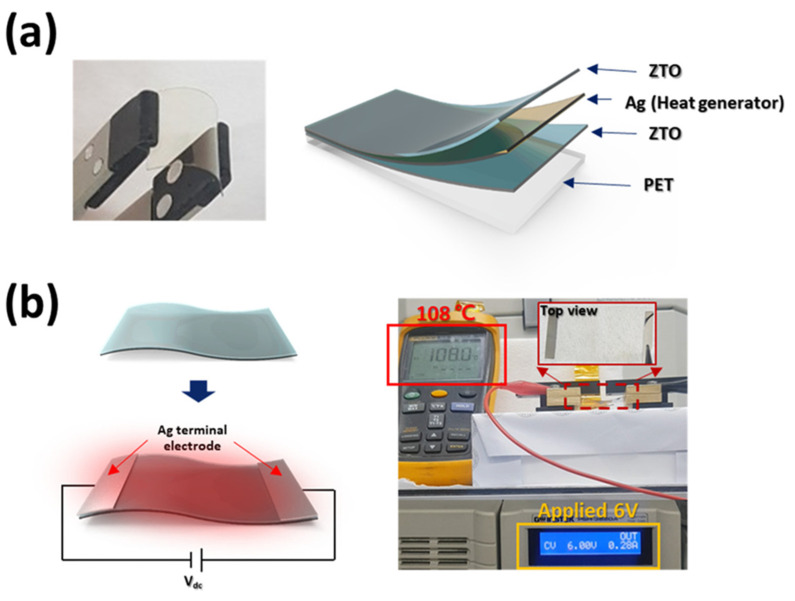
The ZTO/Ag/ZTO multiple-layer thin-film schematic on PET substrates is shown in (**a**). The ZTO/Ag/ZTO-based thin-film heater is shown in (**b**), along with how the heater is operated [7].

A ZTO/Ag/ZTO multilayer thin-film schematic and an image of a flexible, transparent ZTO/Ag/ZTO multilayered thin film coated on a PET substrate are displayed in Figure 4a. Furthermore, as seen in Figure 4b, we constructed a ZTO/Ag/ZTO-based thin-film heater under ideal gas circumstances and verified its functionality [7]. At a concentration of 0.3% oxygen, the ZTO/Ag/ZTO multilayered thin film was formed on substrates made of glass and PET. On-axis RF and DC sputtering were used to create the oxide and Ag layers of the OMO multilayered thin film, respectively. The gas ratio used to create the OMO multilayer is the crucial factor. At 0.3% oxygen concentration, the ZTO oxide layer exhibits the maximum transmittance and the lowest electrical resistance.

In order to improve the performance of thin-film heaters, silver sandwich-like structures integrate a silver layer between functional layers. These designs handle issues including cost, durability, and oxidation while utilizing silver’s exceptional thermal and electrical conductivity. The most recent structural design methodologies and developments are mentioned here. AgNWs are layered between protective coatings and flexible substrates (like PET or polyimide). The nanowires are protected by these coatings against environmental deterioration and mechanical harm. Without sacrificing conductivity, hybrid protective layers composed of graphene oxide, conductive polymers, or oxide films improve transparency and durability.

A flexible transparent conducting film with a sandwiched structure of MXene/AgNW/graphene was presented and effectively used as a transparent heater, according to Pengchang Wang et al. Graphene served as the top protective layer, while MXene served as the intermediate layer between the substrate and AgNW film. In this work, the hybrid TCFs with the unique structure demonstrated optical transmittance, strong adhesion, and good electrical conductivity. The hybrid TCF-based TH showed stability, a quick heat response, and even heat dispersion [25].

Silver sandwich structures employ graphene layers as enhancers or barriers. Conductivity is provided by the silver layer, and oxidation resistance and mechanical stability are guaranteed by graphene. When graphene and silver are combined, they can have synergistic effects including improved flexibility and thermal distribution. The heat transfer gradient was almost five-times greater when graphene was present. In addition to graphene-doped or -layered silver coatings, which are promising materials for heater manufacturing, silver nanoparticles and silver nanofibers are the subject of extensive investigation. One material that shows promise for surface heating is silver-coated graphene. It was discovered that surface topography is crucial to heat energy transfer. Graphene-doped Ag thin films are hence suitable for heat energy transfer and high-performance heater design [26]. Metal oxides such as ZnO or TiO₂ are used in place of thin silver layers to increase thermal stability, adhesion, and transparency. These composites can be precisely constructed using atomic layer deposition (ALD) processes, guaranteeing consistency and reproducibility [27].

Environmental resistance is increased by encasing silver layers in polymers or transparent dielectric coatings. Fluorinated polymers and other advanced encapsulating materials provide superior barrier qualities. Over time, performance may deteriorate due to silver’s susceptibility to oxidation, particularly at high temperatures. Oxidation concerns are decreased by using protective coatings like graphene oxide or transparent polymers.

Due to the high cost of silver, thick or high-purity layers are prohibitively expensive. Silver usage is decreased while functionality is preserved through hybrid designs and nanomaterial techniques. Repeated mechanical flexing or heat cycling can cause sandwich structures to delaminate or crack. Durability is improved with flexible encapsulants and adhesion-promoting interlayers [28]. Stress during heating may result from differences in the thermal expansion coefficients of the surrounding materials and the silver layer. Stress is reduced by using interlayers and compatible substrates with corresponding expansion properties. Maintaining consistency in multilayer systems, particularly at nanoscale thicknesses, requires precision. Layer uniformity is enhanced by sophisticated deposition techniques such as roll-to-roll printing, ALD, and sputtering.

## 5. Electrical Characteristics

One important characteristic of THs is their electrical resistance, which may be measured experimentally in several ways. Uniform thin-film characterization has historically relied on the sheet resistance R_sh_ (Ω sq^−1^), which offers a localized and direct evaluation of electrical characteristics regardless of specimen size. Since the four-point probe method removes the contributions of electrical cables and contributes to the global electrical resistance, it enables a precise measurement of R_sh_. Although this method involves direct touch and may cause local harm, it is typically applied to developing transparent conductive materials (TCMs) such as metallic nanowire networks or thin films. By evaluating a sample’s electrical performance throughout a wide range of conditions, this electrical assessment is highly useful for predicting the sample’s electrical homogeneity. For a homogeneous layer, R_sh_ can be approximated as the ratio of electrical resistivity to layer thickness, which can be determined using an ellipsometer, profilometer, electronic microscope, or Atomic Force Microscopy (AFM). Electrical resistance on materials can also be obtained using simple two-point probes. Generally, metallic coatings produced by evaporation or silver inks are used to fabricate two parallel electrodes at opposing sides of the specimen. A further method for examining electrical homogeneity is the one-probe electrical mapping, which creates a voltage distribution map. Furthermore, the conductivity of AgNW networks has been measured non-contact using terahertz spectroscopy.

## 6. Optical Characteristics

THs’ optical transparency is a crucial characteristic. Transparency in the visible spectrum (390 to 700 nm) is quite good for TCOs like FTO. However, plasmonic absorption causes a sharp drop in their optical transmittances in the near-infrared range. New TCMs, like metallic grids, metallic nanowires, and CNT networks, remain mostly transparent over the entire VIS-NIR spectrum because they permit incident light to pass through the unfilled substrate spaces. Additionally, there are compact and useful devices such as tint meters that offer transparency readings at a specific visible wavelength, typically 550 nm (the wavelength of light at which human vision functions at its best).

**Figure 5:**
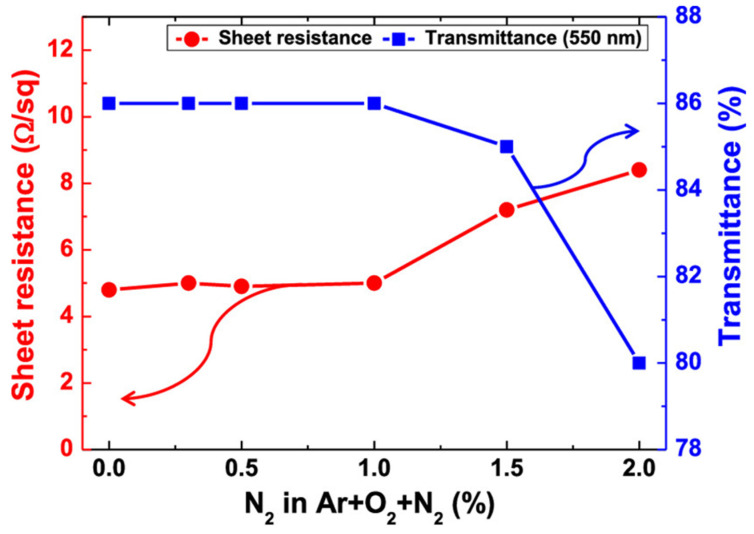
Variations in the N_2_-to-(Ar + O_2_) gas ratio cause changes in the ZTO/Ag/ZTO multilayer thin film’s transmittance and sheet resistance [7].

As the N_2_ to Ar + O_2_ ratio was changed, Figure 5 illustrates the impact of gas composition on the electrical and optical characteristics of a ZTO/Ag/ZTO multilayer thin film on a PET substrate. The ZTO/Ag/ZTO multilayered thin film exhibited a nearly constant sheet resistance from 0% to 1.0%, with a rise beyond that point. It was roughly 5.0 Ω sq^−1^. Moreover, the ZTO/Ag/ZTO multilayered thin film on a PET substrate had almost the same optical transmittance, roughly 86% between 0 and 1.0% nitrogen concentration, but it dropped at 1.0%.

Typically, the optical transparency in the literature is provided at this wavelength of 550 nm or averaged over a typical range of 370–700 nm, which is the visible range. Unfortunately, the authors’ reference to specular (i.e., direct) or total transmittance is not often stated. The diffuse transmission measurement is vitally important.

## 7. Measurement of Temperature

Precise temperature measurement is necessary to achieve fine TH characterization. High temporal and spatial resolutions are anticipated in measurements of the temperature generated by THs as a function of the input voltage or other adjusted parameters. Other typical properties, such as steady-state temperature, heating rate, operating voltage, heat power density, thermal resistance or conductivity, heat transfer coefficient, etc., can be computed and/or defined. A thermocouple that is directly linked to the object or sample being analyzed is a popular method for taking temperature readings. Thermocouples are inexpensive and have a broad temperature range. Various varieties of thermocouples are appropriate for different applications and temperature ranges. The K-type thermocouple is the most frequently reported in TH cases. Resistance measurement is an additional direct-contact method of temperature measurement. Temperature detectors (RTD) rely on the fact that when temperatures rise, conductors’ electrical resistance increases.

## 8. Mechanical Properties

The earliest application of THs occurred during World War II, when hard tin oxide was used to thaw aircraft cockpit windshields, allowing them to fly at higher altitudes. The advancement of nanotechnology and flexible electronics in recent years has resulted in a wide range of applications that demand good mechanical stability. Despite its superior electrical and optical qualities, TCOs cannot be integrated into very flexible systems, as discussed further below. A high temperature deposition renders flexible and transparent polymers unsuitable as substrates because of their low heat resistance. Electrical conductivity in THs must not be affected by mechanical loads, and thermal performance must be stable. Finally, the absorption of TCMs into elastic substrates, as well as their adhesion to diverse substrate types, is another mechanical property issue that may have an impact on the performance of TFH applications. Embedded structures are a viable technique to address adhesion difficulties. Using repeated tape testing and microscopic observations of layer adhesion can identify critical drivers for further study.

## 9. Stability

Electrical, thermal, mechanical, and chemical stability are important factors for TH performance. First, it is vital to ensure that electrical conductivity and heat emitted stay steady over the time that THs are subjected to electrical stressors. Various tests can be conducted, including voltage ramps and plateau cycles with resistance and temperature measurements. Most TH investigations include the “ON-OFF” stability test. A consistent voltage/power is typically applied for hours to ensure that the temperature remains stable. In other circumstances, steadily increasing voltage ramps are used until the heating performance fails, after which lower voltages are used to study potential reversibility. These investigations demonstrated that encapsulating heating materials with protective coatings is a great way to improve stability.

**Figure 6:**
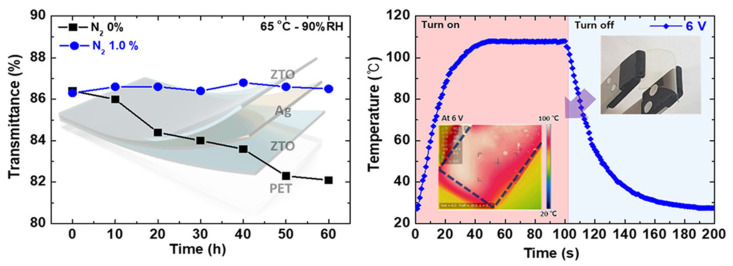
Transparent thin-film heater that defrosts quickly and is flexible and chemically stable [7].

Transparent thin-film heaters with excellent performance and chemical stability are made of Zn-doped SnO_2_/Ag/Zn-doped SnO_2_(ZTO/Ag/ZTO) multilayer thin films [7], which are manufactured on a polyethylene terephthalate (PET) substrate using an optimum N_2_-to-(Ar + O_2_) gas ratio. By adding nitrogen, the ZTO/Ag/ZTO-based multilayer thin film shows improved endurance at high temperatures and humid situations shown in Figure 6.

The ZTO/Ag/ZTO multilayer thin film’s mechanical flexibility is demonstrated by the bending test results, which showed that even after 10,000 bending cycles the sheet resistance did not significantly change. It also displays chemical stability following 100 heating–cooling cycles and a consistent heat distribution at saturation temperature. Therefore, the suggested ZTO/Ag/ZTO-based thin-film heater can be used in construction and automotive applications for front and rear windows. It also displays chemical stability following 100 heating–cooling cycles and a consistent heat distribution at saturation temperature. Therefore, the suggested ZTO/Ag/ZTO-based thin-film heater can be used in construction and automotive applications for front and rear windows.

It is also critical to evaluate the intrinsic heat stability of the substrates used to manufacture a TH, particularly flexible polymers, which are known to limit the stability of flexible THs. Chemical oxidation and aging measurements are typically performed in environmental chambers under controlled humidity and temperature conditions, as well as light irradiation. Generally, the relative humidity is greater than 80%, and the temperature ranges from 45 °C to 90 °C, which covers the majority of the hardest-use circumstances.

## 10. Types of Materials Used in TFHs

Numerous material technologies have been thoroughly examined for use in thermal heaters applications. TCOs were the first class of thin layers and have been studied for many years. These metal oxide thin films have been extensively used in industrial devices and have been researched for a variety of uses, such as transparent electrodes for solar cells or touch displays. Similar to transparent electrodes, research into alternative materials has been spurred by industrial needs for THs. This is due to several factors. For example, TCOs are typically not able to tolerate any mechanical stress because they are ceramic, which means that flexible applications cannot use them.

### 10.1. Materials Based on Metallic Oxides

While metallic-based nanomaterials have been under consideration since roughly the year 2000, other materials, such as TCOs, have been extensively researched for several decades to be employed as transparent electrodes (TEs) or THs [29,30]. When compared to TCOs, they have extra characteristics because many of them have exceptional mechanical flexibility and can be deposited at low temperatures. Since metals have extremely high electrical and thermal conductivities, they do seem like appealing candidates for TH active materials. For example, at room temperature silver has the highest conductivity of all the materials. However, because of considerable light absorption as well as electron scattering at surfaces or interfaces, ultrathin metallic films often do not provide an attractive trade-off between optical transparency and sheet resistance. Therefore, creating metal nanostructures is the only practical option to use metals in TH applications. This can offer intriguing possibilities for controlling photons and electrons to produce mechanical, optical, and electrical characteristics that are not possible with TCOs. Metallic meshes, MNW networks, and metallic grids are the primary metallic patterns that are effective for producing THs. There can be considerable confusion in the literature regarding the distinction between metal grids and meshes. This section will provide a brief overview of the many metallic-based materials that have been explored, outlining their key characteristics, possible applications, and integration with TH devices. We will also touch on developments in resistance, adhesion, stability, physical attributes, and low-cost TH production.

**Figure 7:**
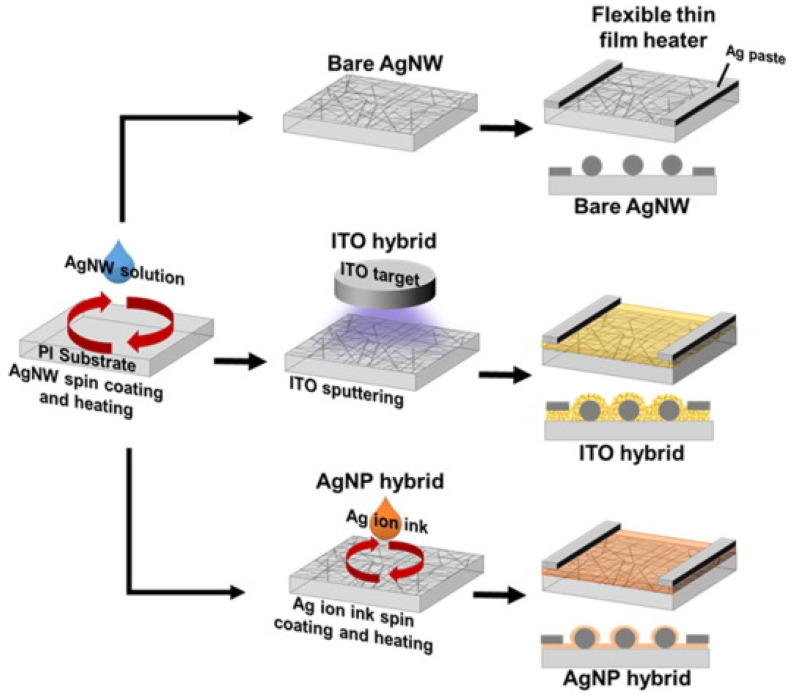
Diagrams showing the film heating and coating procedures for both the bare AgNW and hybrid films on a PI substrate [31].

TCOs were the first kind of TCMs to be created and researched out of all the other varieties. They have a lengthy history of use in a wide range of industrial applications. In particular, ITO thin films have been used to produce THs that defrost airplane windshields. We direct the reader to other studies for further details as they have provided a detailed description of TCOs’ fundamental features. Notably, TCOs’ thin-film shape and structure are crucial to their conductivity. Usually, these thin films have a polycrystalline structure. The flexible thin-film hybrid heaters (f-TFHs) described in Figure 7 is based on hybrid films of Ag nanowire networks (AgNWs) with metals or metal oxides.

AgNWs were created by spin coating polyimide (PI) substrates, and then nanoparticles of silver (AgNPs) and indium tin oxide (ITO) were deposited between and on top of the AgNWs using DC magnetron sputtering and spin coating, respectively. Our experimental findings show that, in comparison to bare AgNWs on a PI substrate, the hybrid films exhibit improved mechanical reliability and a decreased sheet resistance. The heater is formed by the matrix AgNPs and ITO, which also serve as an insulating film to retain the heat produced by the AgNWs. In comparison to the AgNWs, the hybrid TFHs reached a greater temperature and more uniform heating at a fixed υ up to a specific limit. However, local melting of AgNWs happened in and around the center of the TFHs, which is why the hybrid of AgNWs with AgNPs and the TFHs of AgNWs failed to operate over the limit ϕ. 

**Figure 8:**
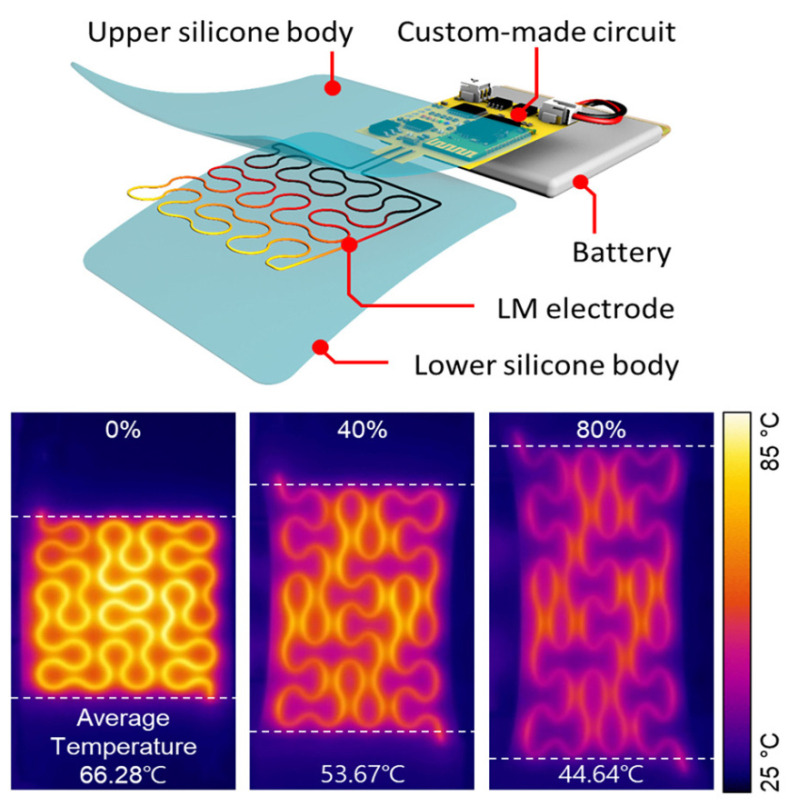
Design of a wearable heater with a liquid metal basis that generates heat continuously under biaxial strain [32].

Because of its many uses, wearable heaters are becoming more and more popular, which means that their tensile stability needs to be improved. In resistive heaters for wearable electronics, multi-axial dynamic deformation with human motion poses a challenge to the precise control and stability of heating. Seongmin Jeong et al. suggested a pattern study for a liquid metal (LM)-based wearable heater circuit control system devoid of deep learning or a complicated structure shown in Figure 8. The wearable heaters were made using the LM direct ink writing (DIW) technique in a variety of designs.

The pattern’s directionality was demonstrated to be a factor that complicates feedback control because of the difference in resistance change according to the strain direction. The pattern’s significance for steady average temperatures with tension was established through the pattern study. Using Peano curves and a sinuous pattern structure, a wearable heater was created to address this problem with the least amount of resistance change independent of the tension direction. Last but not least, the wearable heater with the circuit control system exhibits stable heating (52.64 °C, with a standard deviation of 0.91 °C) in real motion when attached to a human body model.

**Figure 9:**
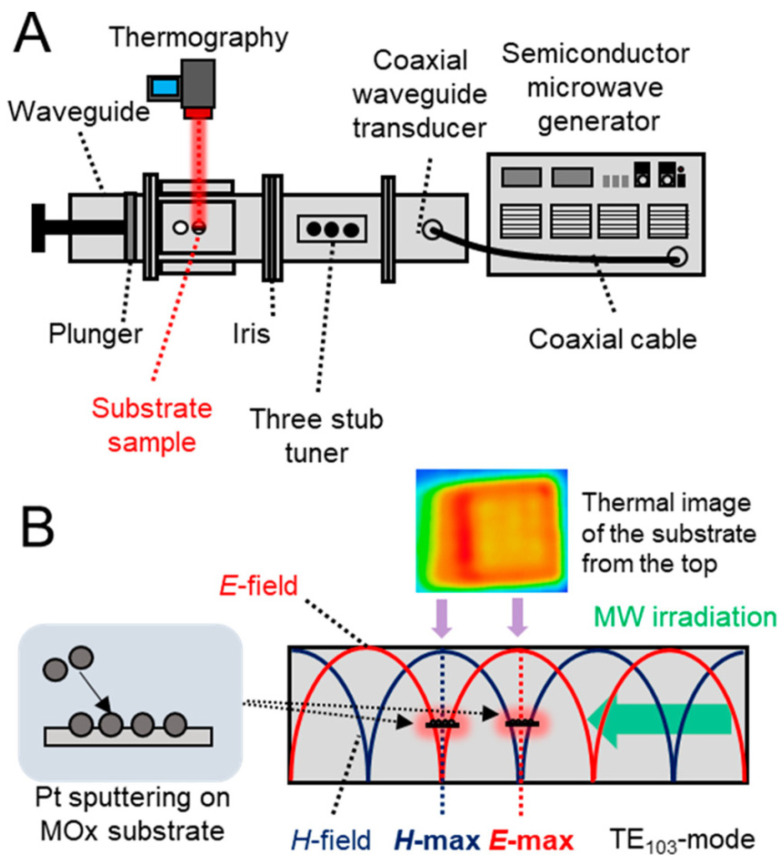
Diagram of the microwave heating system. (**A**) Waveguide cavity resonator with thermography apparatus and a semiconductor microwave generator attached. (**B**) The substrate’s position within the waveguide cavity resonator [33].

The impact of platinum (Pt) nanoparticle sizes and loadings on single-crystal metal oxide (MOx) substrates (Al_2_O_3_, MgAl_2_O_4_, TiO_2_, and SrTiO_3_) with varying relative permittivities and conductivities are demonstrated by Taishi Ano et al.: when Pt NPs were deposited on MOx substrates with low relative permittivities and conductivities, significant heating occurred, suggesting that the most important characteristic is the substrate’s MW transparency shown in Figure 9. Large Pt NP loading amounts were best heated using a magnetic field. According to models of electromagnetic fields, local hotspots were created by the concentration of the electric field between Pt nanoparticles. Furthermore, the frequency of the MWs and the conductivity of the supporting metal influenced the NP/MOx composite’s MW absorption behavior.

The size and loading of the supported metal NPs are the next most crucial factors for assuring effective MW heating by Pt NPs, after the relative permittivity and conductivity of the MOx support. Targeted MW heating of the supported metal catalyst is the outcome of the cooperative action of the metal NPs and MOx support.

### 10.2. Carbon-Based Materials

The first description of carbon-based thin-film heaters appeared in 2007. Numerous studies in the literature have discussed the use of carbon fibers, carbon nanotubes (CNTs), or graphene derivatives in a variety of application areas, such as wearable electronics, thermochromic displays, defogging, anti-icing, and deicing. The majority of the research that has been published discusses the evaluation of non-transparent devices using carbon-based thin-film heaters. Due to their exceptional mechanical qualities, they undoubtedly have considerable flexibility and stretchability, which makes them highly desirable for a variety of heaters; on the other hand, they have low transparency.

Carbon-based THs’ active components can be processed through dry or solution-based methods, with the latter frequently being more appropriate for a variety of substrates (PEN, PET, PC, PI, or cotton fabrics, for example). CNT devices can be made in two ways: either by printing techniques or by percolative networks.

A flexible, transparent, high-performance heater is created using a mix of graphene and dry-spun carbon nanotubes (CNTs), extracted straight from a hyper-vertically aligned CNT forest. Two types of hybrid devices are studied: simple CNT film-heating devices consisting of one or two layers of CNTs, and graphene above or below the CNT film. The electrical, optical, and electromechanical properties of these devices are examined. The outcomes demonstrate the superiority of the hybrid structured film heaters over the basic CNT film heaters.

**Figure 10:**
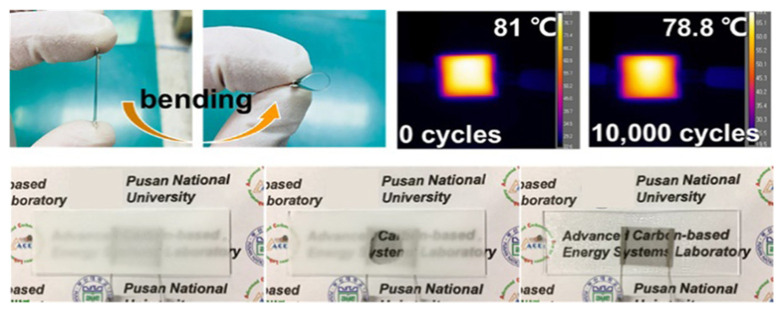
Transparent and bendable heating device utilizing graphene and a dry-spun carbon nanotube hybrid 2D platform [34].

The single-layer CNT sheet/graphene/PET and graphene/single-layer carbon nanotube sheet/PET hybrid heaters reach the highest temperatures of 81 and 85 °C with transmittances of 68 and 71%, respectively shown in Figure 10. The simple single-layer CNT film and double-layer CNT film heaters reach the highest temperatures of forty-eight and sixty-four degrees Celsius with transmittances of 73 and 64% at a wavelength of 550 nm, respectively. The graphene, single-layer CNT sheet and PET heater appear to have excellent mechanical and thermal properties based on the results of the 10,000 bending cycle test. Additionally, the defrost test and portable heating utilizing a 9 V battery demonstrate that the hybrid heater can be used for wearable technology, portable heating, and vehicle defrosting.

**Figure 11:**
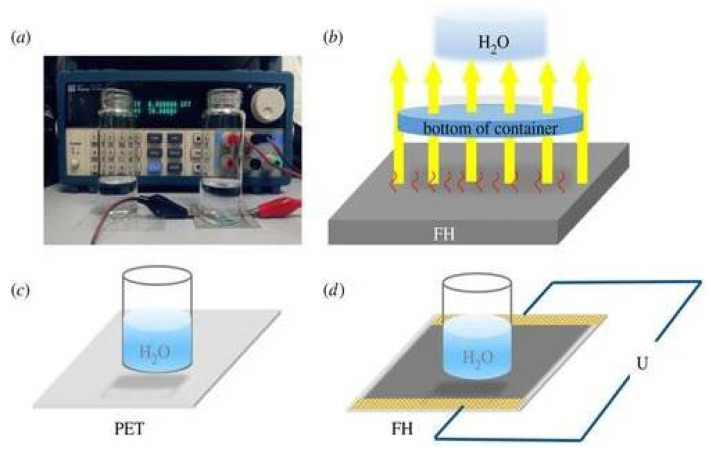
Foldable electrothermal film heaters based on carbon nanotubes that have a high heating rate [35].

It is simple to create outstanding-performance, bendable film heaters using spraying and rod-coating techniques when using transparent conducting films containing carbon nanotubes. The primary findings indicate that the film, when applied via the spraying method following a series of post-treatment procedures using acid and distilled water, exhibits a heating rate of 6.1 °C s^−1^ at 35 V and a sheet resistance as low as 94.7 ohm sq^−1^ with 72.04% optical transmittance at a wavelength of 550 nm shown in Figure 11(a–d). The functions and the gathered experimental data were employed by a mathematical technique called nonlinear fitting. A thorough examination of the formula provides a clear explanation for the relationship between temperature and time. As a result, flexible, transparent heaters based on carbon nanotubes have excellent electrothermal performance and are predicted to find use in a variety of settings, including dining tables, heating materials, and smart windows.

**Figure 12:**
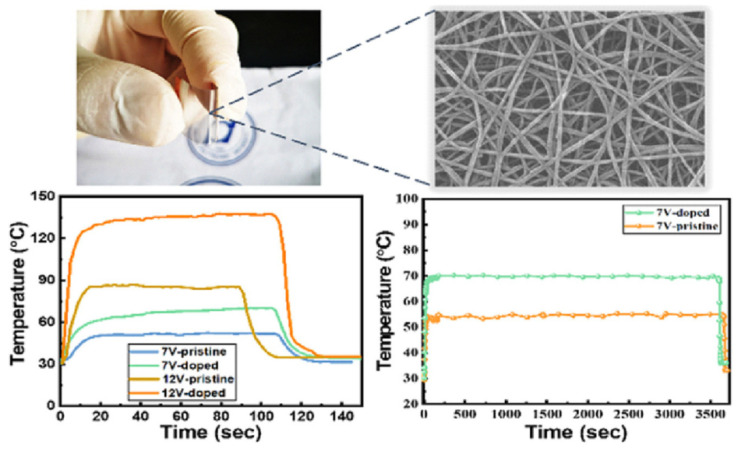
Single-walled carbon nanotube thin films applied by dry method for flexible and transparent heaters [36].

By using floating catalyst chemical vapor deposition (FCCVD) to create high-quality SWCNT thin films shown in Figure 12, Zhao Zhang et al. were able to transfer films directly and dry onto polyethylene terephthalate (PET) as THs. At driving voltages of 7 V and 12 V, respectively, a pristine SWCNT TH with a transmittance of 73.5% demonstrated a respectable heating ability, reaching saturation temperatures of approximately 53 and 85 °C. However, following AuCl3 doping of the same SWCNT sheet, the temperature increases to approximately 70 °C and 139 °C at 7 V and 12 V, respectively. The decreased sheet resistance and film densification caused by AuCl_3_ doping were primarily responsible for the enhanced heating performance. Additionally, we discovered that our SWCNT TH exhibited outstanding mechanical and thermal flexibility.

**Figure 13:**
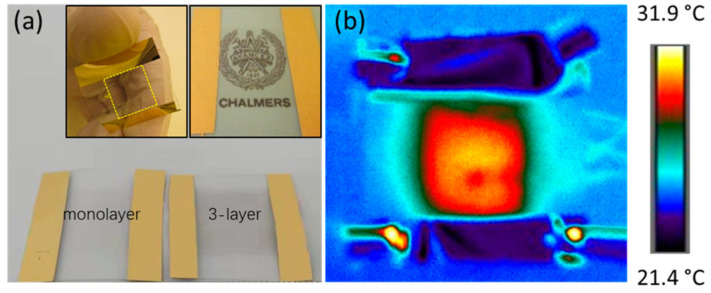
Features of undoped few-layer transparent heaters based on graphene [37].

Clear, effective, and reasonably priced heating elements are required for numerous applications, such as defoggers, sensors, and screens. Because of this, Yong Zhang et al. assessed the electrothermal characteristics of graphene-based heaters [37], including heating/cooling rates and steady-state temperatures as a function of input power density, using one-to-five layers of graphene on flexible and transparent polyethylene terephthalate (PET) substrates shown in Figure 13a,b. For monolayer heaters, the heating/cooling rates had an exponential time dependence with a time constant of somewhat less than 6 s. A convective heat-transfer coefficient of 60 W·m^−2^·°C^−1^ was determined from the relationship between the steady-state temperatures and the input power density, indicating a performance that is significantly better than that of many other types of heaters, such as those based on metal thin-films and carbon nanotubes.

### 10.3. Polymer-Based Materials

Conductive polymers have attracted attention because of their cost-effectiveness, processability, and flexibility/stretchability for thermo-hydrogel production. Few of them, meanwhile, have electrical conductivities appropriate for TH applications.

Recently, the first demonstration of a 100% polymeric TH was performed, marking a breakthrough in the manufacture of THs. Poly(3,4-ethylenedioxythiophene) (PEDOT)-based thin films have been shown by Gueye et al. to be effective THs without the use of conductive fillers or metal. Several conductive polymers were examined: (i) PEDOT doped with ethylene glycol (EG) and polystyrene sulfonate (PSS): PEDOT: PSS-EG, (ii) thin films of PEDOT: OTf treated with diluted sulfuric acid to further improve conductivity, and (iii) PEDOT mixed with trifluoromethane sulfonate CF_3_SO_3_ (OTf): PEDOT: OTf PEDOT: Sul, etc. These conductive polymers are flexible and have good optoelectronic and thermal capabilities. Under severe mechanical stress, the polymer-based TH’s electrical resistance and thermal characteristics do not change.

**Figure 14:**
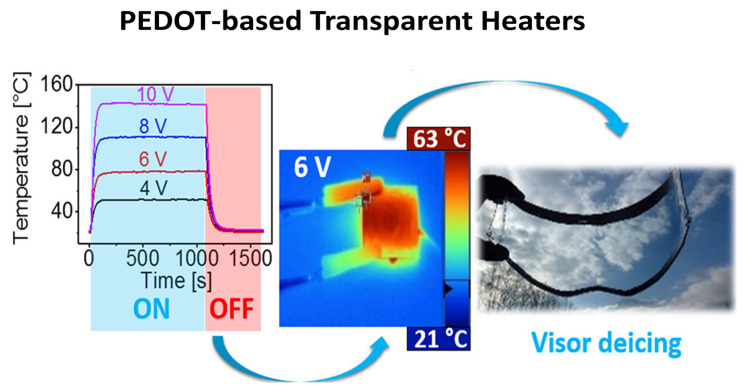
Flexible transparent heaters made of all polymers [38].

Magatte N. Gueye et al. present the first demonstration of all-polymeric flexible transparent heaters (THs). Four poly(3,4-ethylenedioxythiophene) (PEDOT)-based materials’ thin films with varying dopants embedded show low sheet resistances of 57 Ω sq^−1^, which are linked to good transparencies (>87%) and less than 1% of haze shown in Figure 14. When exposed to a 12 V bias, these transparent thin films exhibit exceptional heating capabilities, including high heating rates (up to 1.6 °C s^−1^) and steady-state temperatures above 100 °C. Additionally, extremely high areal power densities of nearly 10,000 W m^−2^ were measured. A thermal model is well fitted to the temperature rise. It is further shown that these novel THs can be effectively integrated for use in visor deicers and thermochromic displays.

**Figure 15:**
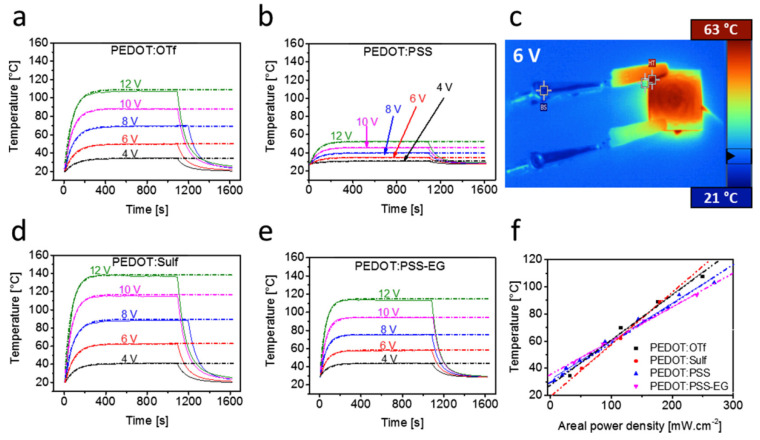
Heating performances of PEDOT-based THs (**a**–**f**). Temperature elevation of PEDOT-based materials deposited by spin coating on glass substrates [38].

Voltages ranging from 4 to 12 V were applied to the four films in order to evaluate the TH capabilities of these PEDOT-based materials (Figure 15a–f. For each of the four materials, the temperature elevation as measured using a K-type thermocouple attached beneath the substrate is shown in Figure 15a,b,d,e). The temperature rise in the film and the connecting wires is depicted in Figure 15c infrared image of a TH. In the case of small-area THs (up to 2.5 cm × 2.5 cm), the sample is heated uniformly throughout. The highest-resistance PEDOT: PSS exhibits the least temperature increase (Figure 15f). PEDOT:OTf and PEDOT: PSS–EG exhibit performances twice as high as PEDOT: PSS at 10 V bias (Figure 15a,e, with 88 and 94 °C, respectively. Superior heating characteristics are displayed by PEDOT: Sulf, which can reach 120 °C at 10 V bias and 138 °C at 12 V bias, respectively, even though it was shown that heat may be generated at temperatures higher than 200 °C.

**Figure 16:**
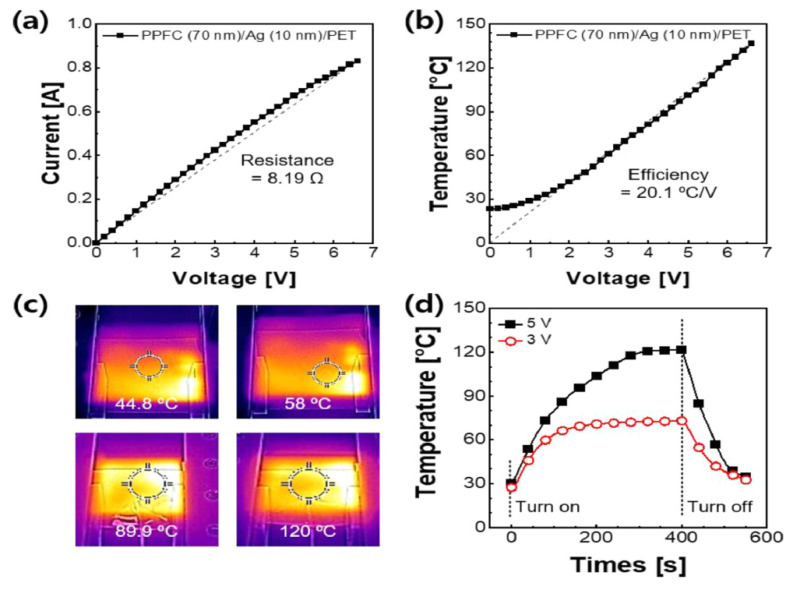
Utilizing the plasma polymer fluorocarbon/silver bilayer thin films and transparent and flexible heaters [39].

Bilayer thin films of silver (Ag) and plasma polymer fluorocarbon (PPFC) are used to produce a transparent and flexible heater shown in Figure 16a–d. The polymer/metal bilayer thin-film structure improves optical transmittance in the visible light range while allowing a simpler procedure than polymer/metal/polymer trilayer thin-film structures. The optimized PPFC/Ag bilayer thin film exhibits a deionized water contact angle of 103.44°, a sheet resistance of 6.67 Ω·sq^−1^, and an optical transmittance of 0.80 at 440 nm. It is possible to employ the heater in flexible and rollable devices without experiencing a significant performance reduction, according to the results of several mechanical flexibility tests. This study sheds light on the application of flexible devices and transparent and flexible heaters in building external walls, and automotive glasses to avoid frosting and fogging.

**Figure 17:**
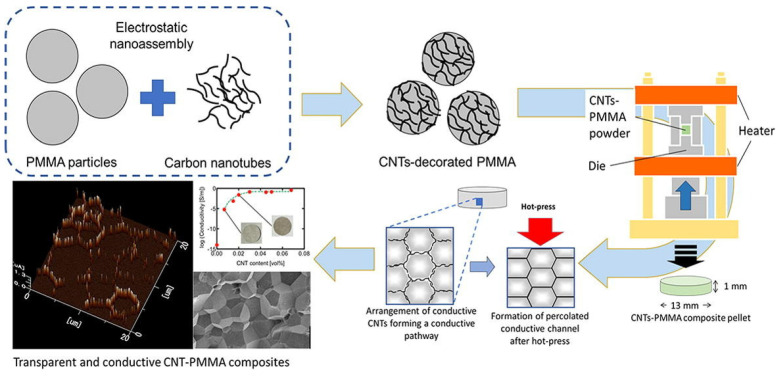
Conductive polymer composites that are transparent for use as heaters [40].

There has been a growing interest in the controlled production of high-tech, transparent carbon-based composite materials for use in small, light devices. This work describes the possible synthesis of composite pellets made of poly (methyl methacrylate) (PMMA) and carbon nanotubes (CNTs) that demonstrated exceptional optical transparency and strong electrical conductivities shown in Figure 17. Even with a modest percentage of CNT integration (0.0068–0.068 vol%), the homogenous decorating of CNT onto PMMA particles produced by the electrostatic assembly technique resulted in the generation of conductive channels. Using a scanning probe microscope equipped with contact current imaging, the conductive channels that developed at the pressed interface of the CNT–PMMA pellets were verified. This work offers a promising avenue for producing carbon-based composite materials using a technology inspired by powder metallurgy, which can be applied to the production of transparent, conducting polymers and lightweight products. These latest advancements, which are based on polymer thin films with an inherent high conductivity, pave the way for new, extremely effective, and entirely organic THs.

**Figure 18:**
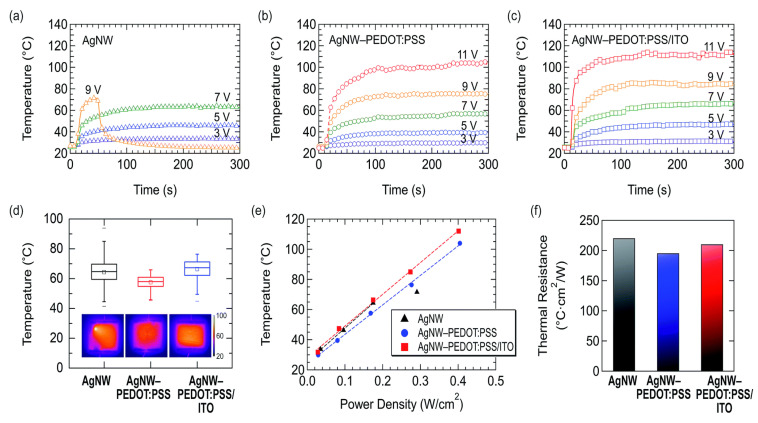
(**a**–**f**) Bendable transparent film heaters using a ternary mixture of conducting polymer, conductive oxide, and silver nanowire [41].

A number of requirements, including excellent optical transparency, low electrical resistance, uniform heating, and operational stability under varied environmental conditions, must be met in order to produce TFHs with high performance and practicality. It is difficult to meet every need at once, though, because optical transmittance and electrical resistance have trade-offs. In this work, we report on flexible thin-film heaters (TFHs) that are made of a ternary composite of silver nanowire (AgNW), conducting polymer (poly[3,4-ethylenedioxythiophene]: polystyrene sulfonate [PEDOT: PSS]), and a thin conductive oxide (indium tin oxide [ITO]) layer shown in Figure 18a–f. These TFHs outperform pristine AgNW-based TFHs in terms of the highest temperature of heating (>110 °C), operational stability, mechanical flexibility, and optical transmittance (95% at 550 nm). The AgNW-PEDOT: PSS/ITO TFHs operate steadily while submerged in water, demonstrating exceptional environmental resilience. Using a device simulator, we examined the development of Joule heating to analyze the fundamental mechanisms underlying the improved efficiency of the AgNW–PEDOT: PSS/ITO TFHs. We discovered that the improvement resulted from both increased heat dissipation with PEDOT: PSS and ITO and decreased electrical resistance. The optical transmittance, sheet resistance, and thermal stability for various types of TFH materials from literature shown in Table 1. We hope that the continued development of useful flexible TFHs will benefit from our investigation and findings.

Because of the high temperatures of operation and rapid heating/cooling rates, thermal stability is essential for THs and is associated with the possible deterioration of the active substances or surrounding materials. Measuring resistance to electricity under thermal stress (heat ramps or plateaus) without using any electrical power aside from a very tiny current for the resistance measurement is one method to investigate this. Investigating the inherent heat durability of the substrates used to create a TH is also crucial, particularly when it comes to flexible polymers, which are known to restrict the stability of flexible THs. Measurements of chemical oxidation and aging are typically conducted in environmental chambers, where regulated temperature and humidity are simultaneously applied along with light irradiation [48].

## 11. Methods to Improve the Efficiency of THs (Oxidation Resistance, Moisture Sensitivity, Adhesion Enhancement, and Stability)

An additional crucial element in ensuring that transparent conducting films (TCFs) can be applied to transparent flexible heaters (TFHs) is the adherence of conducting films to the substrate. The adherence between conductive coatings and the substrate is weak for TCFs made using the traditional coating method. It may result in minor surface imperfections in real-world application processes, such as scuffs, fractures, porosity, etc., which would cause the coating to become non-uniform and raise resistance in certain areas. This could lead to hotspots and the heater breaking down [51]. To effectively address the aforementioned problems, we integrated the conductive coating into waterborne polyurethanes (WPUs), which have excellent flexibility, high optical transparency, excellent abrasion, and chemical resistance [52]. This allowed us to create a high-performance TCF with an embedded structure for TFHs. Thus, WPU is intended to be utilized as TFHs’ freestanding substrate [53].

Oxidation resistance and moisture sensitivity have a major impact on thermal heater longevity because they affect how long the heater will last and function in different environmental circumstances. This is how durability is affected by each. During operation, thermal heaters, particularly those composed of metallic materials, are subjected to high temperatures. Materials are more likely to oxidize at higher temperatures, forming oxide coatings that can impair their mechanical and thermal characteristics. Over time, oxidation can result in pitting, scaling, or embrittlement, which lowers the heater’s efficiency. Materials utilized for heating elements, such as ceramic coatings, nickel alloys, and stainless steels, must have high oxidation resistance. Resistance can be increased by protective surface coatings or treatments (such as aluminization).

To preserve structural integrity and performance, heaters exposed to corrosive gases (such as sulfur compounds) or oxygen-rich atmospheres need to have an improved oxidation resistance.

Moisture can erode insulation materials and create corrosion in metallic heaters, which can result in failure or short circuits.

According to Chang-Lae Kim et al., wearable heaters based on silver nanowire (AgNW) were evaluated for durability, electrical performance, and thermal performance. Furthermore, the WR coating demonstrated excellent performance and endurance in shielding AgNW electrodes from corrosion in challenging conditions with high humidity. Consequently, the improved smart wearable heaters had high electrical, thermal, and mechanical performance, and the AgNW electrodes were extremely durable, thanks to the structural coating that used materials with properties like shape memory (self-ironing or drip-dry) and water-repellent properties (self-cleaning) [54].

According to Jianyu Chen et al., a successful technique for enhancing the oxidation resistance of copper nanowires (Cu NWs) is to coat them with nickel using a one-pot production method. Understanding the connection between the thickness of the Ni coating layer and the characteristics of NWs is crucial because Ni is far less conductive than Cu. The oxidation temperature is higher in Cu–Ni NWs with a thicker Ni layer, though. The ideal Cu–Ni NWs had a beginning oxidation temperature of 270 °C and a Ni-layer thickness of roughly 10 nm. They were made using a Cu^2+^/Ni^2+^ molar ratio of 1/1. The resulting transparent conducting films have a sheet resistance of 300 Ω sq^−1^ and a transmittance of 76%. When it comes to heating and defrosting, the flexible heater made of such premium Cu–Ni NW sheets performs admirably [55].

Tao wang et al. [56] reported that high-performance TFHs can be made from the ultra-low-roughness, high-adhesion TG/TCNT/PEDOT-WPU TCFs with an embedded structure. Tannic acid (TA), a plant-based polyphenol that is environmentally friendly, has been shown to act as a dual non-covalent stabilizer for carbon nanotubes, or CNTs, and a stripping medium in the process of microfluidizing graphite to graphene. Waterborne polyurethane (WPU) film was mixed with conductive nanocomposites (TA-functionalized grapheme/TA-functionalized CNT/PEDOT:PSS; TG/TCNT/PEDOT) to create a high-performance transparent flexible heater (TFH) with an embedded structure. These films showed excellent mechanical strength (the sheet resistivity stayed nearly constant after a thousand bending cycle test for the bending radius of 10 mm), low root mean square (rms) roughness (approximately 0.37 nm), favorable optical transmittance, and favorable sheet resistance (T = ca. 80% at 550 nm, Rs = 62.5 Ω/sq.). These properties make them perfect for use as transparent heaters with a high thermal efficiency. The temperature rose quickly and achieved a steady state in 20 s, with a maximum temperature of 116 °C. Furthermore, following repeated heating–cooling tests and a long-term stability test, no temperature variation was noted, suggesting that TG/TCNT/PEDOT-WPU TCFs can be utilized as high-performance TFHs. It is anticipated that these TFHs will work with smart windows, portable heating, smart wearables, and car defrosting.

**Figure 19:**
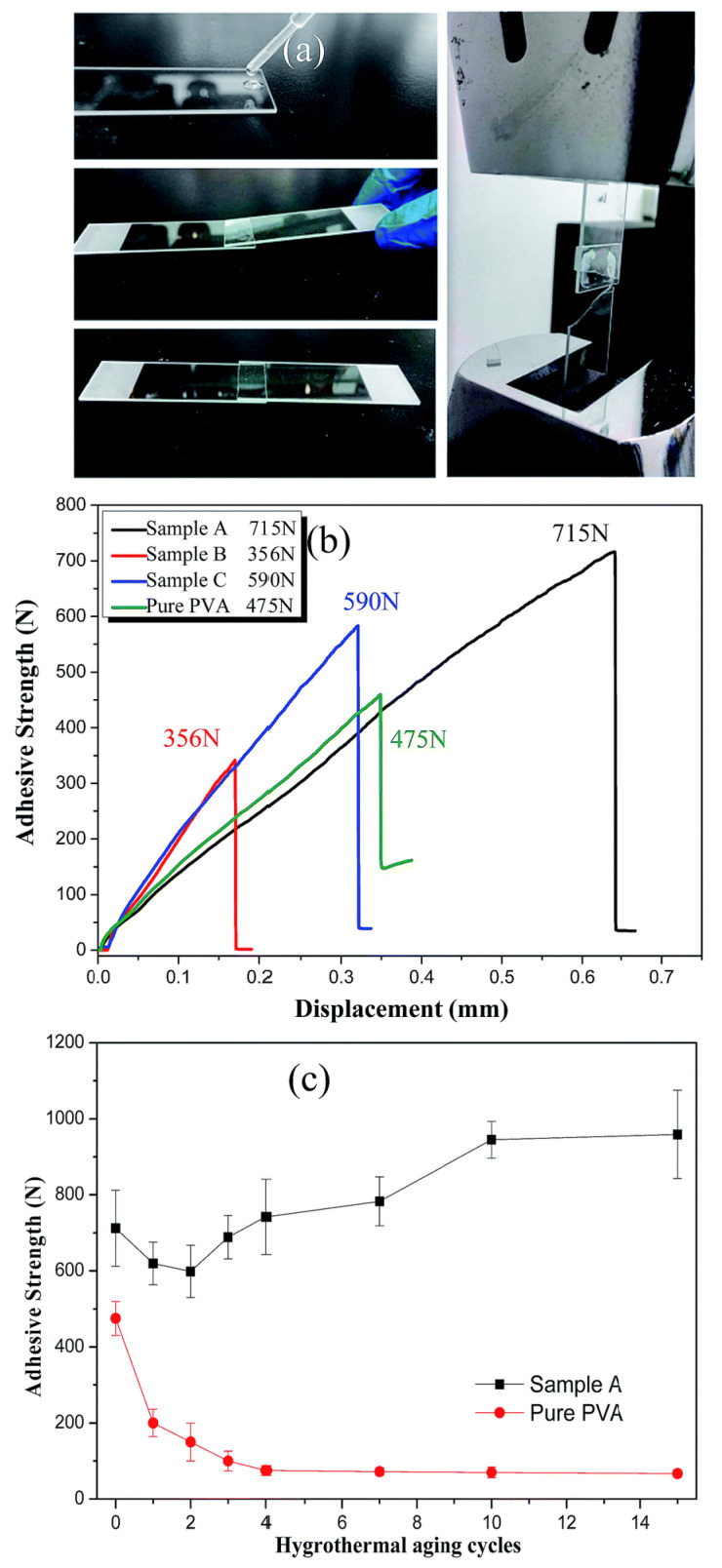
(**a**) Streamlined procedures for performing the adhesion test; (**b**) adhesive strength for every sample as a function of displacement; and (**c**) adhesive strength following cycles of hygrothermal aging [57].

For common applications, it is envisaged that silica/PVA hybrids can preserve PVA’s adhesive strength. However, when 59 nm silica nanoparticles are prepared using the conventional Stöber’s process for hybrid compounds, phase separation is observed when the silica content exceeds 36.5 wt%. While combining nanoparticles with polymer has been shown to increase adhesive strength in certain tests, these studies used very little inorganic material. The lap sheer strength of the overlap joint for each of the silica/PVA hybrid samples was determined in order to comprehend the impact of silica particles on the adhesive strength, as indicated in Figure 19a. Sample A exhibits an exceptional adhesive strength when measured against the adhesive strength of pure PVA (475 N, 1.52 MPa). As seen in Figure 19b, Samples A (715 N, 2.29 MPa) and C (590 N, 1.89 MPa) exhibit excellent adhesive strengths when compared to the adhesion capacity of pure PVA (475 N, 1.52 MPa). Furthermore, hygrothermal aging cycles were used to replicate humidity and hot/cold conditions to assess the adhesive strength’s longevity. The adhesive strengths of Sample A and pure PVA were determined following various hygrothermal aging cycles, as indicated in Figure 19c. In Sample A, the adhesive strength drops in the first two cycles from 715 ± 90 N to 619 ± 55 N (24 h into the first cycle) and 598 ± 68 N (48 h into the second cycle). PVA’s partial dissolving owing to its hydrophilic characteristics could be the reason for this.

**Figure 20:**
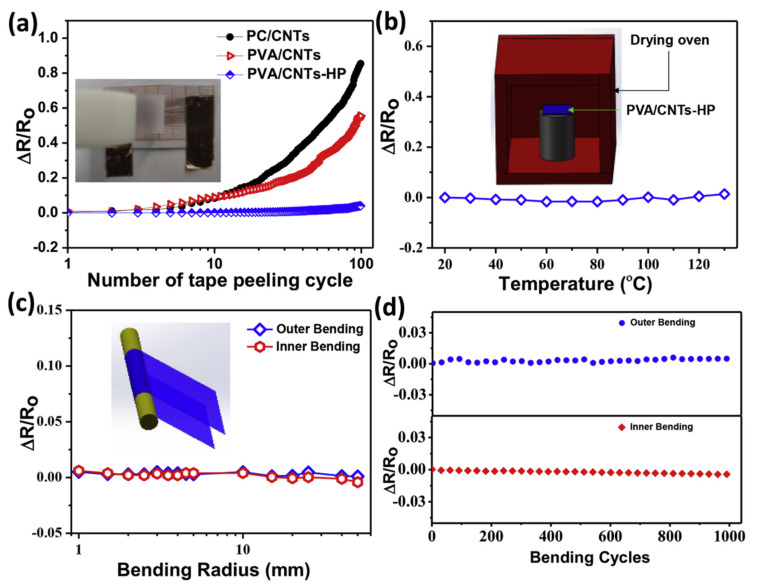
(**a**–**d**) Flexibility and interfacial adhesion of poly(vinyl alcohol) film with single-walled carbon nanotubes [58].

Since high interfacial adhesion is thought to be essential for TFHs to withstand the harsh environments encountered in real-world applications, it is a major source of worry. To determine the CNT conductive layer’s interfacial adhesion ability with different substrates, peeling-off tests using 3M Scotch tape were conducted in this work (inset in Figure 20a). As illustrated in Figure 20a, 3M Scotch tape can readily peel off the loose CNT network on the PC substrate’s surface, increasing the PC/CNT film’s sheet resistance by over 100% after 100 peeling-off cycles (Figure 20d). Due to the high contact with TCE or TFH film, such weak interfacial adhesion makes PC/CNT film difficult to utilize in real-world situations. Because PVA is partially submerged in the CNT layer, spin coating the CNT network onto PVA film helps to increase its adherence with the substrate. Even after only ten peeling-off cycles, the resulting PVA/CNT shows a noticeable improvement in resistivity, although it still has low peeling resistance. The PVA/CNTs-HP film exhibits a remarkable improvement in interfacial adhesion between the conductive layer and substrate, as demonstrated by its sheet resistance during the repeated Scotch tape peeling test for up to 100 cycles (Figure 20c). This phenomenon may be attributed to the complete embedding of the CNT network through the hot-pressing process. The fundamental assurance for PVA/CNT-HP film utilized as a TFH with a steady conductivity and Joule heating performance is the strong interfacial adhesion. However, because of the frequent temperature changes in real-world applications, the resistivity stability versus TFH ambient temperature is similarly significant. Because of the strong thermos-oxidative stability of the CNT conductive layer, PVA/CNTs-HP displays outstanding resistivity stability even at elevated temperatures of around 130°C, as shown in Figure 20b.

**Figure 21:**
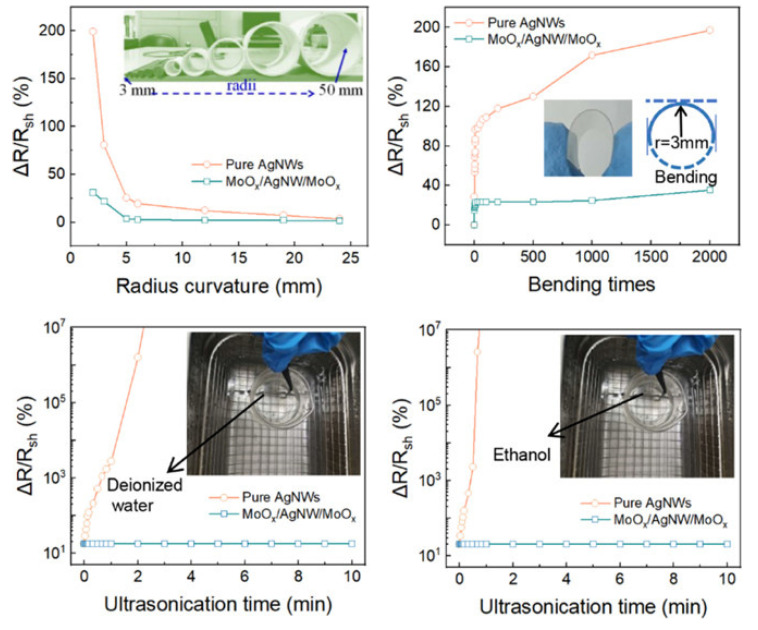
MOO-encapsulated Ag nanowire flexible transparent conductors with enhanced adhesion and conductivity [59].

Using the full-solution method without high-temperature annealing, a novel transparent conductive conductor made of a MoOx and silver nanowire (AgNW) network on a flexible polyethylene terephthalate (PET) substrate is created, offering better adhesion and decreased resistance at the same time. With 89.2% optical transmittance and 12.5 Ω/sq low sheet resistance, a MoOx/AgNW/MoOx multilayer is created under optimal circumstances, exhibiting a substantially higher optoelectronic performance compared to that obtained from ITO shown in Figure 21. In contrast to pure AgNW films, there is very little change in the sheet resistance following the tape and ultrasonication tests, indicating that MoOx has a very strong adherence to the PET substrate after encapsulation. Additionally, the multilayer film shows outstanding resilience against acid degradation and mechanical bending.

Without a vacuum process or a high-temperature annealing phase, a straightforward procedure is intended to improve the adhesion and photoelectric capabilities of AgNWs on PET. A minimal sheet resistance of 12.5 Ω/sq, high transmittance of 89.2%, good mechanical flexibility, and strong adherence to the PET substrate are the results of successfully inserting an AgNW network into the MoOx layers. Bending experiments show that the very thin MoOx layer and the flexibility of AgNW considerably increase the flexibility of MoOx/AgNW/MoOx composites. We created FTHs to show how the electrodes could be used. The heater responds quickly and exhibits outstanding thermal resistance. The MoOx/AgNW/MoOx composite films found in the described TTCEs had exceptional flexibility, strong adhesion, great conductivity, and high transparency.

**Figure 22:**
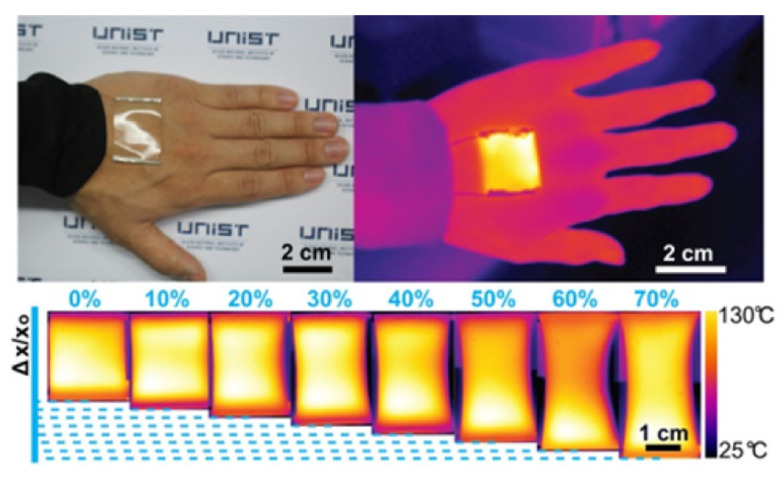
Utilizing nanotrough networks of metal glass with outstanding mechanical strength and thermal stability. Stretchable and transparent electrodes are used as wearable heaters [43].

Due to its excellent electrical conductivity, corrosion resistance, and elastic limit, metallic glasses (MGs) show promise for use in wearable electronics [2]. Nevertheless, their uses in transparent electrodes or wearable electronics have not yet been thoroughly investigated. The CuZr MGs in the form of nanotrough networks to create transparent and stretchy electrodes show in the Figure 22. Stretchable elastomeric substrates are just one of the many substrates to which MG nanotroughs can be transported after being created by the electrospinning and co-sputtering processes. The resultant MG nanotrough network is initially applied as a stretchy transparent electrode, exhibiting exceptional mechanical and optoelectronic robustness (3.8 Ω/sq at 90% transmittance) and chemically stable behavior under humid and hot environments.

The resultant transparent, stretchable electrode and heater have exceptional mechanical robustness against deformations like folding and stretching, as well as exceptional chemical stability against oxidations and optoelectronic qualities. Numerical research provides substantial support for the notion that the CuZr nanotrough network’s extraordinary stretchability is mostly due to the enormous elastic limit inherent in MG. Examples of applications for this MG heater include a skin-attachable thermal patch with a wireless temperature control via a smartphone and a transparent defroster for car side-view mirrors. We think that a viable approach to next-generation electronics is the usage of MGs for wearable electronics and automotive applications.

**Figure 23:**
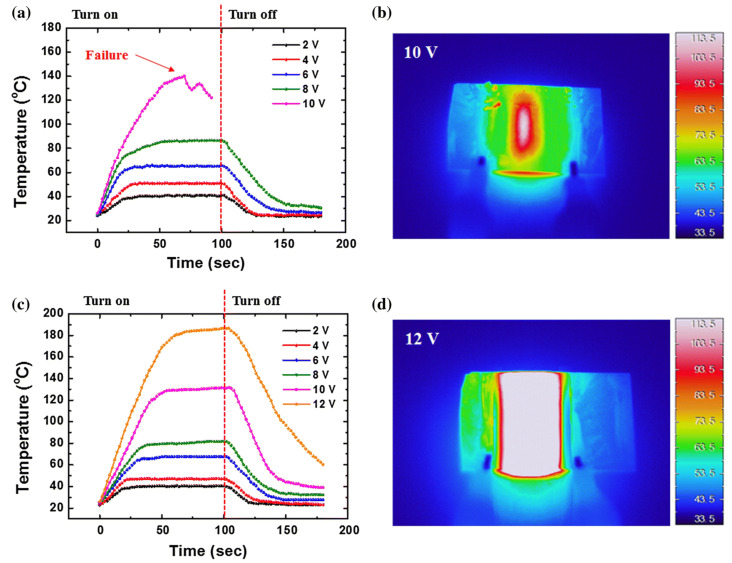
(**a**–**d**) SWCNT–Ag nanowire hybrid for improved electrical stability in a transparent stretchable film heater [61].

For transparent and flexible electrode materials to be used in stretchable electrical devices, they must be mechanically stable [60]. This work produced composite films of silver nanowire (Ag NW) and single-walled carbon nanotubes (SWCNT) as electrode materials to enhance the anti-electromigration and thermal stability of transparent stretchable film heaters shown in Figure 23. The mechanical and anti-electromigration characteristics of SWCNT–Ag NW hybrid films were systematically examined by varying the addition ratio of SWCNT–Ag NW suspensions (Fig 23, a-d). The 75:1 SWCNT–Ag NW composite film demonstrated superior thermal stability, enhanced anti-electromigration characteristics, and a low sheet resistance of 62.3 Ω/sq with an optical transmission of 83.4% in comparison to the pristine Ag NW film. Moreover, after 1000 stretching cycles under tensile stress, the identical composite film made on VHB substrate only displayed a 23.2% increase in relative sheet resistance. Additionally, even after 1000 stretching cycles at a peak strain of 200%, the stretchable film heater with the 75:1 SWCNT–Ag NW composite electrode showed better thermal and mechanical stability.

**Figure 24:**
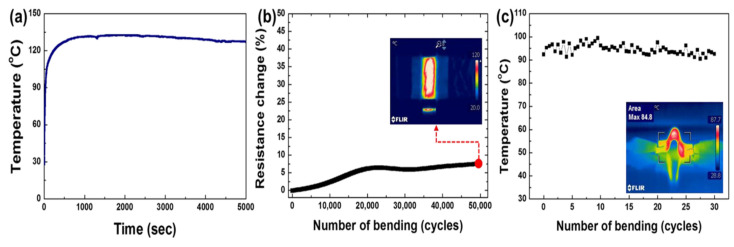
(**a**–**c**) The flexible heater that is transparent and strong mechanically is created by combining Ag nanowires with conductive polymers [62].

Using a solution-processed inverted layer-by-layer processing technique, a composite electrode consisting of silver nanowires (AgNWs) and a conductive polymer (CP) embedded in a colorless polyimide (cPI) film was produced to create a transparent and highly flexible heater. The resulting CP-AgNW/cPI heater proved to be mechanically stable during long-term cyclic bending to a curvature radius of 500 μm for up to 50,000 cycles, with increases in resistance of less than 10% shown in Figure 24. The remarkable flexibility was caused by the electrodes being entirely implanted at the polymer’s surface, according to a roughness assessment. The manufactured electrode reached a peak temperature of more than 200 °C in less than 40 s after being heated quickly from ambient temperature to steady-state temperature.

## 12. Recent Developments in Transparent Thin-Film Heaters

A simple technique for creating transparent, flexible, and high-performing thin-film heaters (TTFHs) was presented by Suhyeon Han et al. [63]. The method relies on Cu–Ag/indium tin oxide bilayer transparent thin-film electrodes (TTFEs) with both nano- and microstructures that are based on nanostructured micromesh (NSMM).

**Figure 25:**
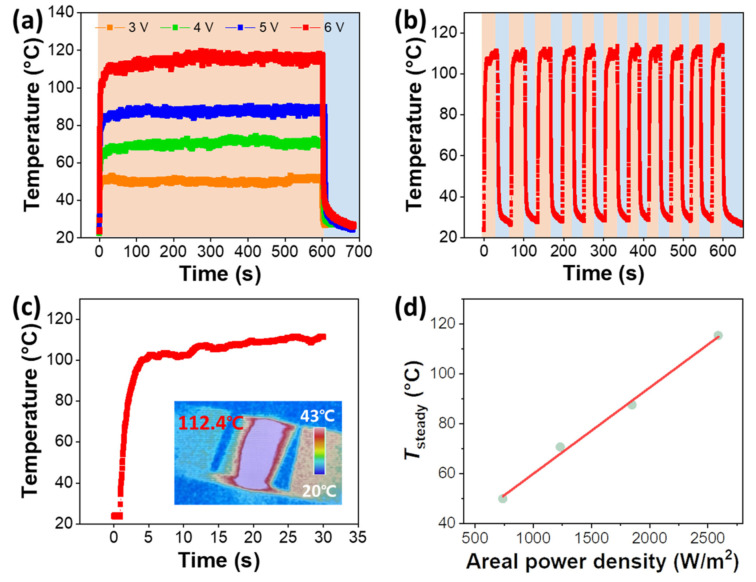
(**a**–**d**) Electrodes having nanostructured matter micromesh Cu–Ag ultrathin films that are very flexible and transparent for efficient thin-film heaters [63].

Our methodology fabricates NSMM TTFEs with a high optical transmittance (about 80.1%) and low sheet resistance (2.1 Ω/sq) by combining pulsed laser ablation and Ar-assisted thermal evaporation processes. The good figure of merit values (51.8 × 10^−3^ Ω^−1^) were obtained for the NSMM TTFEs by varying the microhole spacing. Additionally, these electrodes exhibit exceptional flexibility and durability, as demonstrated by a resistance change of approximately 4%–18% in 200,000 cycle cyclic bending testing shown in Figure 25. With their quick response times (3.97 s at a constant temperature of 87.5 °C under low-voltage operation (5 V)) and infrared shielding capabilities, the NSMM TTFEs can be utilized as high-performance TTFHs. Our findings imply that the Cu–Ag alloy-based NSMM TTFEs are transparent electrodes with great promise for a range of uses in wearable technology, flexible electronics, and smart windows.

**Figure 26:**
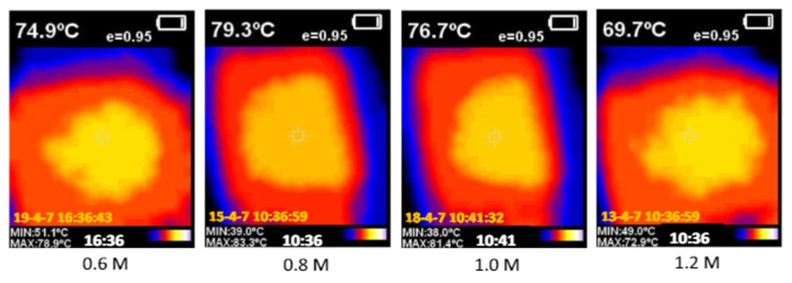
Al-doped SnO_2_ thin films for a transparent heater: impact of the concentration of tin (II) chloride [64].

The initial concentration has been adjusted from 0.6 to 1.2 M at intervals of 0.2 M in order to examine the impact of SnCl_2_.2H_2_O concentration on the characteristics of the sample and heater performance. The main cassiterite SnO_2_ phase is seen in the samples at diffraction angles of 27.14, 34.41, 38.55, 52.26, and 54.28° shown in Figure 26. Four basic vibrational modes are seen in the sample at approximately 80.97, 780.50, 1080.13, and 2518.38 cm^−1^, in that order. The morphology displays octahedral grains, the largest of which corresponds to the sample with a concentration of 1.0 M. The concentration of SnCl_2_.2H_2_O dramatically affects optical transmittance; in the visible range, the sample prepared at 0.8 M has the maximum transmittance.

**Figure 27:**
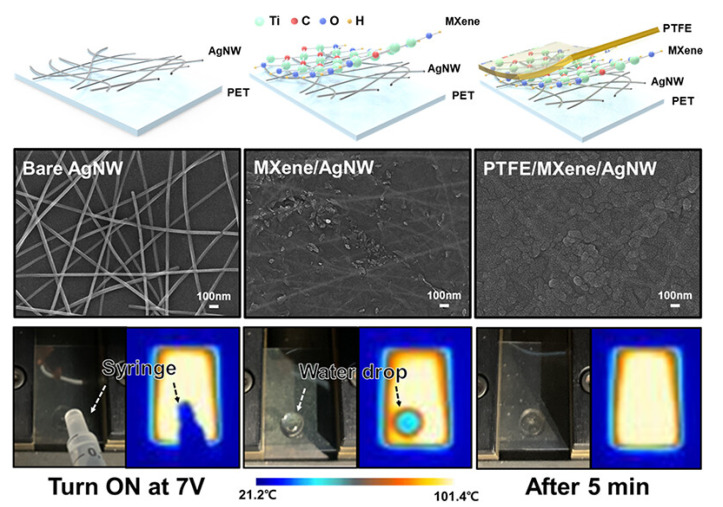
A 2D MXene and 3D Ag nanowire hybrid electrodes with transparent polytetrafluoroethylene thin films as heaters[65].

Conformal polytetrafluoroethylene (PTFE) passivation thin films were created by Seong-Won Kim et al. by utilizing magnetron sputtering at room temperature. The nonpassivated bare MA-composite electrode showed a significantly higher sheet resistance than the PTFE-passivated electrode. Additionally, due to the harsh testing conditions, moisture and impurities severely oxidized the MXene layer, which resulted in rapid degradation of the electrode flexibility and electromagnetic interference shielding efficiency (SE). Even after the 85 °C–85% relative humidity (RH) test, the MA-composite electrode with PTFE passivation exhibited consistent sheet resistance, transmittance, flexibility, and EMI SE shown in Figure 27. The PTFE-passivated MA-composite electrode-based thin-film heaters (TFHs) demonstrated better stability and dependability than nonpassivated MA-electrode-based TFHs, suggesting that the sputtered PTFE film successfully stopped moisture and contaminant penetration of the MA-composite electrodes.

**Figure 28:**
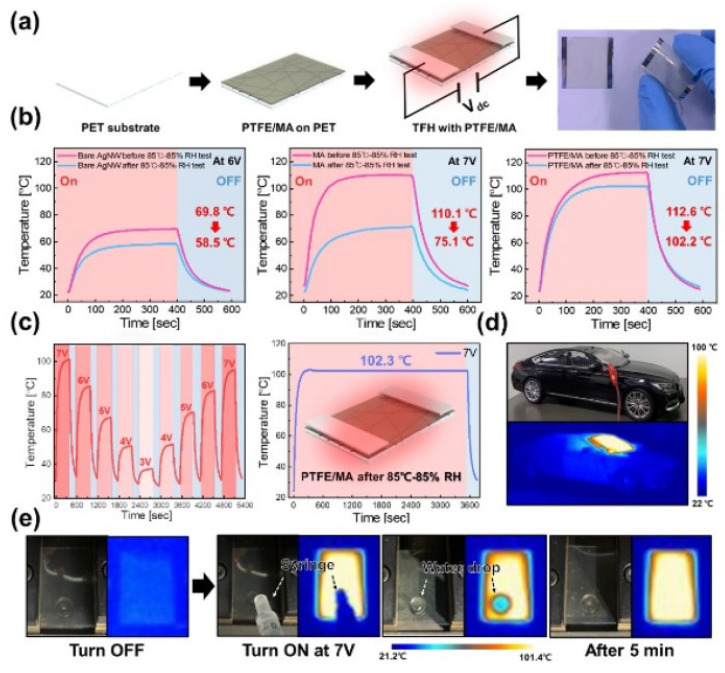
The method for creating flexible TFHs (**a**). (**b**) Analysis of the heat profiles for the bare AgNW and MA-composite before and after the test at 85 °C and 85% relative humidity. (**c**) Testing the stability and heating/cooling of TFHs repeatedly. (**d**) A picture and matching infrared image of TFHs mounted on a minicar model’s front windshield. (**e**) Images and related infrared pictures demonstrating the hot PTFE-passivated MA-composite-based TFH’s water-droplet test [65].

Furthermore, the PTFE-passivated MA-composite-based TFH demonstrated exceptional durability and dependability under repeated heating and cooling circumstances as well as constant heating above 100 °C for one hour during the 85 °C–85% RH test shown in Figure 28a–d. Thus, the RF-sputtered PTFE-passivated MA-composite thin-film electrodes can be employed to develop future energy-efficient and multifunctional TFHs for use in wearables, cars, smart buildings, and bio-patches. They can also be suitable for blocking contaminants and moisture penetration of MA-composite electrodes operating in harsh environments.

**Figure 29:**
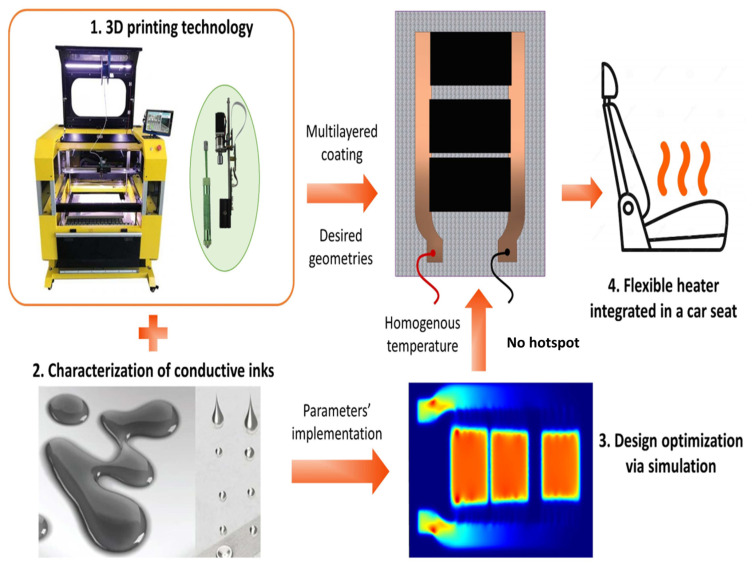
Flexible electronic coatings made by 3D printing: an innovative approach to adaptive heating fabrics for automotive uses [66].

A brand-new generation of heated automobile seats were built using additive manufacturing (AM) and creative, adaptable electronic design. Several layers of conductive materials were applied to the fabric substrate in an attempt to maintain a constant temperature of 41–43 °C in a quick response time without overheating shown in Figure 29. The printing quality was validated by morphological characterizations based on SEM observations, which also showed the suitability of the chosen composite inks for the AM process. Experiments and analytical mixing rules were used to conduct electrical and thermal characterizations to determine the relevant properties of the materials. These results were not only necessary for the FEM implementation using the COMSOL software, but they also made the design strategy easier to understand.

An ultra-flexible and transparent heater based on a composite sheet of Ag nanowire (AgNW) networks and polymers was disclosed by Xue Chen et al.. The AgNWs networks were coated using a commercial polymer called polycarbon waterborne polyurethane resin (PUD 3039). Because of its exceptional permeability, the PUD polymer firmly envelops the nanowires to create a tight, stable conducting network. The AgNWs/PUD composite electrodes have outstanding chemical and mechanical stability. The composite films’ surface roughness was significantly optimized. The 5 × 5 cm^2^ heater was constructed and tested. The results demonstrated long-term stability, great repeatability, and a high saturation temperature of 141 °C at a low voltage of 5 V [67].

Shihui Yu showed how to embed silver nanowires (AgNWs) into a thin UV-curable resin film to create an ultra-flexible transparent conductive hybrid film. This AgNW/UV-curable resin hybrid film performs exceptionally well in terms of transparency (optical transmittance: 86.4%) and conductivity (sheet resistance: 9.9 Ω/sq.). The material continues to exhibit exceptional electrical performance and minimal decay even after being bent 1000 times at a 100 μm curvature radius or even put through an ultrasonication test. After 60 h at high temperatures (85 °C) and high humidity (85%), the hybrid film exhibits environmental durability and does not significantly degrade in performance. It is shown that the hybrid film can be successfully incorporated into a transparent film heater. The heaters’ temperature can rise to 121 degrees due to the effective Joule heating [68].

**Figure 30:**
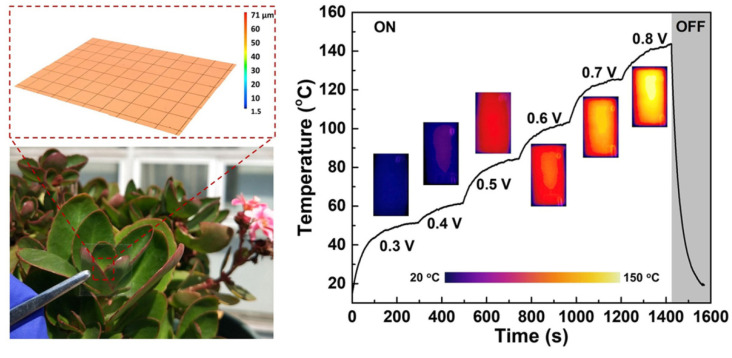
Transparent electrodes made of copper/silver composite mesh with little reflection for high-performance, low-voltage transparent heaters [70].

According to reports, Shihui Yu et al. demonstrated a novel kind of ultra-flexible, biodegradable TCF built from earthly and green materials shown in (Figure 30). By using a straightforward sol–gel transition, copper nanowires (CuNWs) are embedded into chitosan to produce TCFs. The produced TCFs have good electrical and optical characteristics (83.7% at 14.1 Ω/sq. sheet resistance) [69].

Shuang-Shuang Li et al. developed a type of Cu/Ag hybrid mesh transparent conducting electrodes (TCEs) that have low reflection on glass substrates using an easy-to-use technique, and they were successfully used to create high-performing transparent heaters. Cu/Ag composite mesh TCEs with superior optical and electrical properties were effectively prepared by combining laser direct writing with PVP coating-assisted localized removal of sputtered metal layers. These TCEs demonstrated a low average reflectance of 5.71% while retaining a high average transmittance of 86.49% and a low sheet resistance of 0.43 Ω/sq. With an ultra-low applied voltage of 0.8 V, the transparent heater based on the as-obtained Cu/Ag composite mesh TCE demonstrated an efficient and quick heating capability, as demonstrated by reaching a steady-state temperature of around 131 °C in 100 s [70].

For the first time, Xinzuo Huang et al. showed how to create a conductive network for electrical actuation by substituting transparent metal mesh for conductive infill. Solution casting is used to insert the self-cracking aluminum mesh in the transparent shape memory polyimide film (Alm@TSMPI). The Alm@TSMPI is a novel kind of transparent and flexible heater with the benefits of a high steady-state temperature and quick reaction. The Alm@TSMPI is an electric actuator that is both active and malleable based on its changing stiffness properties. When stimulated with electricity, it returns to its initial shape in 13 s [71].

## 13. Market Size for Transparent Film Heaters in 2024: Prospects for Growth and Outlook

The estimated USD million in 2022 will give rise to USD million by 2031 in the worldwide transparent film heater market, which is expected to grow at an impressive CAGR of 2024–2031.

Transparent film heater sales in North America are predicted to grow at an impressive compound annual growth rate (CAGR) of USD million from 2022 to USD million in 2031.

About a percentage of revenue in 2021 came from the top three vendors in the world. The transparent flexible heater, which held a percentage of the global market for transparent film heaters in 2021, is predicted to reach million USD by 2031, rising at a revised impressive CAGR from 2022 to 2031, in light of the economic shift brought on by COVID-19 and the influence of the Russia–Ukraine War. Flexible transparent heaters are bendable and are made mostly of polyimide, with an optically transparent substrate with a conductive coating.

Prominent companies in the flexible transparent heater sector comprise GEOMATEC, Nissha, Transparent Products, Heatron, Super Optics Development, Honeywell, Thin Film Devices, Linepro Controls, and Optical Filters, among others. The top three manufacturers among them promised a % global supply in 2022.

In terms of consumption regions, North America, Europe, and Asia Pacific accounted for the majority of the volume of flexible transparent heaters sold in 2022. Furthermore, China is expected to draw greater interest from investors and industry insiders because of its significant role in the global market for flexible transparent heaters.

The stringent energy conservation standards and growing popularity of electric vehicles in North America and Europe are driving up demand for transparent heaters. Because of the expanding electronics and automotive industries in the Asia Pacific area, particularly China, the market is predicted to be dominated by this region. The market circumstances and trends in the transparent heater industry are thoroughly examined in this research study on the transparent heater market. The growing need for energy-efficient heating solutions across a range of industries is likely to propel the market’s growth at a compound annual growth rate (CAGR) of % during the projected period. The increased use of transparent conductive materials to create heaters, developments in nanotechnology to provide effective heating solutions, and the increasing need for transparent heaters in the automotive, electronics, and aerospace industries are some of the market trends for transparent heaters [72].

The growing demand for energy-effective heating solutions across a range of industries and the developments in transparent conductive materials are expected to propel the market for transparent heaters in the years to come.

## 14. Conclusions and Future Scope

Significant facets and current advancements concerning transparent thin-film heaters have been discussed in this review. Novel materials like metal oxide nanoparticles, carbon nanotubes (CNTs), graphene, metal nanowires, and metal meshes can be used to create transparent heaters in creative ways. There is a detailed discussion of the performance aspects of heaters, including the achievable heating rates and thermal figure of merit values for the examples found in the literature. The demand for low resistance without sacrificing transmittance, a trade-off issue shared by all transparent conductors, is the largest obstacle to heater development. Together with a neutral color, a sheet resistance in the range of about 2–10 Ω/sq, transmittance >80%, and haze <10% are deemed ideal.

While all of these innovative technologies have succeeded in meeting prototype standards, further work is undoubtedly necessary to guarantee stability, cost-effectiveness, and reproducibility at the industrial level. Reproducibility is primarily dependent on stable large-scale production equipment and multiscale characterization methodologies, as well as the identical physical and chemical purity of the starting materials (CNTs, graphene, MNWs, and polymers). Aspects of stability are also quite significant and should be addressed while keeping the intended operating circumstances in mind. The old technology TCOs that have been refined over time are still largely used in the fabrication of THs today. Because this technique is a dependable and well-established process, it will continue to be used in many current and future applications. But THs will also have other qualities (determined haze factor, flexibility, flexibility, and low-cost deposition methods) thanks to several current technological advancements. We anticipate that there will not be just one victor among them. Instead, several novel TH technologies will soon find industrial uses.

We summarized the most current advancements, tactics, and market size growth and projection in this review, along with the goals of enhancing the characteristics, stability, integration, and developments of the transparent thin film for thermal heaters.

## Figures and Tables

**Table 1:** The optical transmittance, sheet resistance, and thermal stability for various types of TFH materials.

TFH Materials	Optical Transmittance (%)	Sheet Resistance (Ohm/Square)	Heating Temperature (°C)	Refs.
SnO_2_ (FTO)/Ag/FTO	83.04%	8.00	117 °C	[42]
CuZr metal glass	90	3.8	180	[43]
Ag NWs	94.5	12.5	178	[44]
Copper nanowires/polyurethane	66.5	22.3	120	[45]
Au–Ag–Au tri-layer film	85	10.42	150	[46]
NiO@Ag NW	86	9.4	266	[47]
RgO thin film	40.80	5160	127.5	[48]
Al,Ga co-doped ZnO thin-film	90	142.5	132.3	[49]
ZnO/Ag/ZnO	90.2	6.2	168	[50]

## Data Availability

MDPI Research Data Policies.
